# Prevalence of DSM-IV Alcohol Abuse and Dependence: United States, 1992

**Published:** 1994

**Authors:** Bridget F. Grant, Thomas C. Harford, Deborah A. Dawson, Patricia Chou, Mary Dufour, Roger Pickering

**Affiliations:** Bridget F. Grant, Ph.D., Ph.D., is the chief of the Biometry Branch, Division of Biometry and Epidemiology, NIAAA, Bethesda, Maryland. Thomas C. Harford, Ph.D., is the director of the Division of Biometry and Epidemiology, NIAAA. Deborah A. Dawson, Ph.D., is a mathematical statistician (biomedical), Division of Biometry and Epidemiology, NIAAA. Patricia Chou, Ph.D., is a mathematical statistician (biomedical), Division of Biometry and Epidemiology, NIAAA. Mary Dufour, M.D., M.P.H., is the acting deputy director of NIAAA. Roger Pickering, M.S., is a computer specialist, Division of Biometry and Epidemiology, NIAAA

## Abstract

For the first time, results are presented on the prevalence of alcohol abuse and dependence in the United States in 1992, according to the most recent psychiatric classification of alcohol-related disorders from the *Diagnostic and Statistical Manual of Mental Disorders, Fourth Edition* (DSM–IV). More than 7 percent of adults surveyed met DSM–IV criteria for 1-year alcohol abuse, alcohol dependence, or both. Males were almost three times more likely than females to meet the criteria for alcohol abuse and/or dependence; however, the male-to-female ratio was lowest in the youngest age group among nonblack respondents, suggesting that the rates of these disorders in nonblack females may be catching up.

This Epidemiologic Bulletin presents prevalence and population estimates of alcohol abuse and dependence in the United States for the year 1992. The definitions for these alcohol-related disorders were based on the most recent criteria from the *Diagnostic and Statistical Manual of Mental Disorders, Fourth Edition* (DSM–IV) (American Psychiatric Association [APA] 1994). Prevalence defines the weighted percentage of respondents classified with a DSM–IV diagnosis, and population estimate refers to the number of people in the United States receiving a DSM–IV diagnosis of alcohol abuse, alcohol dependence, or both. One-year prevalence estimates were derived from self-reports of symptoms of alcohol abuse and dependence on the 1992 National Longitudinal Alcohol Epidemiologic Survey (NLAES). The figures presented in this bulletin are the first estimates of DSM–IV alcohol abuse and dependence to be reported at the national level.

## Background and Procedures

Prevalence and population estimates of alcohol abuse and dependence were based on the 1992 NLAES, a nationwide household survey sponsored by the National Institute on Alcohol Abuse and Alcoholism (NIAAA). Field work for the study was conducted by the Bureau of the Census. For the NLAES, direct face-to-face interviews were conducted with 42,862 respondents, 18 years of age and older, in the contiguous United States and the District of Columbia. The household-response rate for the NLAES was 91.9 percent, and the person-response rate was 97.4 percent.

The NLAES featured a complex multistage design (Massey et al. 1989). Primary sampling units (PSU’s)^1^ were stratified according to sociodemographic criteria and were selected with probability proportional to size. Approximately 2,000 PSU’s were in the 1992 NLAES sample, 52 of which were self-representing—that is, selected with certainty. Within PSU’s, geographically defined secondary sampling units, referred to as segments, were selected systematically for each sample. Oversampling of the black population was accomplished at this stage of sample selection. The decision to oversample the black population was based on the higher observed rates of alcohol-related disease (i.e., liver cirrhosis) in this group.

Segments then were divided into clusters of approximately four to eight housing units, and all occupied housing units were included in the NLAES. Within each household, one randomly selected respondent, 18 years of age or older, was selected to participate in the survey. Oversampling of young adults, 18–29 years of age, was accomplished at this stage of the sample selection to include a greater representation of this heavy drinking population subgroup. This subgroup of young adults was sampled at a ratio of 2.25 percent to 1.00.

Because of the complex survey design of the NLAES, variance estimation procedures that assume a simple random sample cannot be employed. Research has shown that clustering and stratification of the NLAES sample may result in standard errors much larger than those that would be obtained with a simple random sample of equal size. To take into account the NLAES sample design, all standard errors of the prevalence estimates presented here were generated using SUDAAN (Research Triangle Institute 1994), a software program that uses appropriate statistical techniques to adjust for sample design characteristics.

## DSM–IV Classification

The 1992 NLAES included an extensive list of questions designed to assess the presence of symptoms of alcohol abuse and dependence during the 12-month period preceding the interview. We developed these questions, in part, to operationalize the DSM–IV criteria for alcohol-related disorders. Although the DSM–IV classification was not published until the second quarter of 1994, all of the specific diagnostic criteria for alcohol abuse and dependence were known prior to beginning the NLAES interviews (APA 1991) and therefore were incorporated into the final survey instrument in their entirety. What was not known prior to taking the NLAES into the field was which of the diagnostic criteria would be relegated to abuse and dependence categories. However, once all relevant DSM–IV diagnostic criteria were incorporated into the NLAES, computer algorithms could be designed to represent accurately the placement of the criteria within abuse and dependence categories consistent with the finalized diagnostic criteria. Correspondence of the DSM–IV criteria with individual NLAES questions is shown in the sidebar.

**1992 National Longitudinal Alcohol Epidemiologic Survey**

DSM–IV Alcohol Abuse and Dependence Diagnostic Criteria and Associated Questionnaire Items

***Diagnostic Criteria For Alcohol Abuse***

**Diagnostic Criterion: Continued to drink despite social or interpersonal problem caused by drinking**

**Questionnaire Item:**

- Continue to drink even though you knew it was causing you trouble with your family or friends.

Diagnostic Criterion: Recurrent drinking in situations where alcohol use is physically hazardous*

**Questionnaire Items:**

- Drive a car, motorcycle, truck, boat, or other vehicle after having too much to drink.
- Get into a situation while drinking or after drinking that increased your chances of getting hurt—like swimming, using machinery, or walking in a dangerous area or around heavy traffic.

Diagnostic Criterion: Recurrent alcohol-related legal problems*

**Questionnaire Item:**

- Get arrested or held at a police station because of your drinking.

Diagnostic Criterion: Recurrent drinking resulting in failure to fulfill major role obligations at work, school, or home*

**Questionnaire Items:**

- Get drunk or have a hangover when you were supposed to be doing something important—like being at work, school, or taking care of your home or family.
- Get drunk or have a hangover when you were actually doing something important—like being at work, school, or taking care of your home or family.

***Diagnostic Criteria for Alcohol Dependence***^1^

**Diagnostic Criterion: Tolerance**^2^

**Questionnaire Items:**

- Find that your usual number of drinks had much less effect on you than it once did.
- Find that you had to drink much more than you once did to get the effect you wanted.

**Diagnostic Criterion: Withdrawal syndrome**^3^ **or withdrawal relief/avoidance Questionnaire Items:**

- Have any of the following experiences happened when the effects of alcohol were wearing off [Pause], several hours after drinking [Pause], or the morning after drinking? For example, did you *ever*:
  - Have trouble falling asleep or staying asleep.
  - Find yourself shaking when the effects of alcohol were wearing off.
  - Feel depressed, irritable, or nervous.
  - Feel sick to your stomach or vomit when the effects of alcohol were wearing off.
  - Have a very bad headache.
  - Find yourself sweating or your heart beating fast when the effects of alcohol were wearing off.
  - See, feel, or hear things that were not really there.
  - Have fits or seizures when the effects of alcohol were wearing off.
- Take a drink to get over any of the bad aftereffects of drinking.
- Take a drug other than aspirin, Tylenol™, or Advil™ to keep from having a hangover or to get over the bad aftereffects of drinking.
- Take a drink to keep from having a hangover or to make yourself feel better when you had one.

Diagnostic Criterion: Drinking larger amounts over a longer period of time than intended*

**Questionnaire Items:**

- Start drinking even though you decided not to or promised yourself you would not.
- End up drinking more than you meant to.
- Keep on drinking for a much longer period of time than you had intended to.

Diagnostic Criterion: Persistent desire or unsuccessful efforts to cut down or control drinking*

**Questionnaire Items:**

- Want to stop or cut down on your drinking.
- Try to stop or cut down on your drinking but found you could not do it.

**Diagnostic Criterion: Important social, occupational, or recreational activities given up or reduced in favor of drinking**

**Questionnaire Items:**

- Give up or cut down on activities that were important to you in order to drink—like work, school, or associating with friends or relatives.
- Give up or cut down on activities that you were interested in or that gave you pleasure in order to drink.

**Diagnostic Criterion: Great deal of time spent in activities to obtain alcohol, to drink, or to recover from its effects**

**Questionnaire Items:**

- Spend so much time drinking that you had little time for anything else.
- Spend a lot of time being sick or with a hangover from drinking.
- Spend a lot of time making sure that you always had alcohol available.

**Diagnostic Criterion: Continued to drink despite knowledge of having a persistent or recurrent physical or psychological problem caused or exacerbated by drinking Questionnaire Items:**

- Continued to drink even though you knew it was making you feel depressed, uninterested in things, or suspicious or distrustful of other people.
- Continued to drink even though you knew it was causing you a health problem or making a health problem worse.

*In order for the criterion to be positive, either: (a) two or more symptoms must have occurred at least once, or (b) one or more symptoms must have occurred at least twice during the past year.1Dependence diagnoses can be specified with physiological dependence (i.e., evidence of either tolerance or withdrawal) or without physiological dependence (i.e., no evidence of either tolerance or withdrawal).2Tolerance need have occurred only once during the past year for the criterion to be positive.3Two or more symptoms of withdrawal must have occurred at least twice during the past year for the criterion to be positive.

According to DSM–IV, a diagnosis of alcohol abuse requires that a person exhibit a maladaptive pattern of alcohol use, leading to clinically significant impairment or distress, as demonstrated by at least one of the following: (1) continued use despite a social or interpersonal problem caused or exacerbated by the effects of drinking; (2) recurrent drinking in situations in which alcohol use is physically hazardous; (3) recurrent drinking resulting in a failure to fulfill major role obligations; or (4) recurrent alcohol-related legal problems. A diagnosis of alcohol dependence requires that a person meet at least three of seven criteria defined for dependence in any 12-month period (see sidebar).

In the *Diagnostic Statistical Manual of Mental Disorders, Third Edition, Revised* (DSM–III–R) (APA 1987), the duration criteria associated with abuse and dependence specify that some of the symptoms of the disorder must occur continuously during a month or repeatedly over a longer period of time. Unlike that of the DSM–III–R, the duration criteria of the DSM–IV abuse and dependence categories are associated with the individual diagnostic criteria and not the categories of abuse and dependence per se. The duration criterion for both alcohol-related disorders defines the repetitiveness with which certain diagnostic criteria must occur during a 12-month period for these criteria to be considered positive. As shown in the sidebar, the duration criteria for abuse and dependence are not associated with all diagnostic criteria and are defined by qualifiers, such as “recurrent,” “often,” and “persistent” desire or unsuccessful “efforts.”

To satisfy the duration criterion for abuse, a respondent must have experienced two or more symptoms of an abuse criterion associated with a duration qualifier at least once during the past year, or alternatively, at least one symptom of that diagnostic criterion must have occurred at least twice during the past year. For those abuse criteria not associated with a duration qualifier, a related symptom need only have occurred once in the past year to be counted as positive toward an abuse diagnosis.

Similarly, to satisfy the duration criterion for dependence, at least one symptom of a diagnostic criterion associated with a duration qualifier must have occurred at least twice over the course of the year preceding the interview, or alternatively, two or more symptoms related to these criteria must have occurred at least once during the same time period.

The diagnosis of dependence presented in this bulletin was qualified further in an important way. Because the withdrawal criterion of alcohol dependence is defined in DSM–IV as a withdrawal syndrome (i.e., a cluster of symptoms), at least two symptoms of withdrawal, which met the duration criterion, had to occur during the past 12 months. It should be noted, however, that withdrawal is not required for a DSM–IV diagnosis of dependence. The DSM–IV diagnostic category for dependence could be specified further by evidence of physiological dependence (i.e., evidence of either tolerance or withdrawal, including drinking to relieve or avoid withdrawal) or no physiological dependence (i.e., no evidence of tolerance and withdrawal).

## Summary of Findings

Table 1 presents the 1-year prevalence rates, standard errors, and population estimates of DSM–IV alcohol abuse and dependence by age, sex, and ethnicity. The DSM–IV abuse and dependence groups formed by the 1992 NLAES were mutually exclusive. Respondents classified as alcohol abusers did not meet criteria for alcohol dependence; however, those who met criteria for dependence were classified as to whether they also met the criteria for alcohol abuse. Hierarchically, the DSM–IV does not allow a diagnosis of abuse in the presence of dependence, and thus all respondents classified in this bulletin as alcohol dependent with and without abuse would receive only a formal diagnosis of dependence. The purpose of disaggregating respondents classified as dependent with and without abuse merely was to provide more detail concerning the diagnostic status of respondents classified as alcohol dependent.

The 1-year prevalence of combined alcohol abuse and dependence in the NLAES sample was 7.41 percent, representing 13,760,000 Americans (table 1). Slightly more respondents were classified as alcohol dependent (4.38 percent) than as abusing alcohol (3.03 percent). Among those respondents meeting DSM–IV diagnostic criteria for dependence, the greatest proportion also met criteria for alcohol abuse. The predominance of the dual abuse-dependence diagnosis was generally consistent for each age, sex, and ethnic subgroup of the population. The majority of respondents with alcohol dependence diagnoses also were classified with physiological dependence (4.25 percent) in contrast to no physiological dependence (0.13 percent) (data not shown).

One-year prevalence of alcohol abuse and dependence combined was much greater among males (11.00 percent) than females (4.08 percent). Prevalence also was greater among nonblacks (7.68 percent) than among blacks (5.28 percent) (data not shown). Rates for nonblack males and females exceeded the rates for their black counterparts by 27.18 percent and 32.23 percent, respectively.

Prevalence rates of alcohol abuse and dependence were higher among respondents under 45 years than among those 45 years or older, regardless of sex or ethnicity (table 1). For males, the prevalence rate in the youngest age group (18 to 29 years) was 22.07 percent. The rate decreased approximately 50 percent among 30-to-44-year-old males (10.65) and was reduced to 1.18 among those 65 years and older. For females, the highest prevalence rate also was found in the youngest age group (9.84 percent), with the rates falling steadily to 0.27 percent in females 65 years and older. Possible explanations for the decline in alcohol abuse and dependence rates with age may include faulty recall accompanying increasing age, lower survival rates among alcoholics, and various response styles. Alternately, the age gradient may reflect a true cohort effect; that is, that alcohol abuse and dependence are more prevalent among the younger generation of Americans.

**Figure 1:**
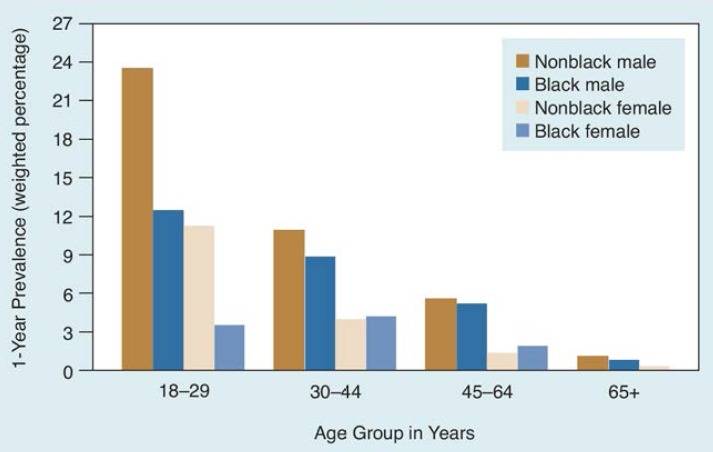
Prevalence of DSM-IV alcohol abuse and dependence by age, sex, and ethnicity: United States, 1992.

Ethnic groups showed striking patterns of age-related 1-year prevalence rates of alcohol abuse and dependence (figure 1). Among the youngest males, the prevalence rate in nonblacks (23.48) was 1.9 times greater than in blacks (12.33). In the remaining age groups, the rates for non-blacks and blacks converge, with a slight predominance among nonblacks. The patterns for nonblack and black females were similar to those of males, execept the black female rate exceeded the non-black female rate among 30-to-64-year-old groups.

Although alcohol abuse and dependence were greater among males than among females, there was evidence of convergence of the rates between the sexes in the youngest age groups (table 2). The male-to-female ratios (i.e., male rate divided by the female rate) were lowest in the 18-to-29-year-old group. However, when the male-to-female ratio was examined separately for each ethnic group, it was clear that the rate converged among the youngest age groups only among nonblacks. In contrast, the male-to-female ratio was much lower among blacks in the 30-to-64-year-old groups. Thus, alcohol abuse and dependence were more prevalent in the younger age groups, particularly among nonblack females.

## Discussion

More than 7 percent of adults surveyed met DSM–IV criteria for 1-year alcohol abuse, alcohol dependence, or both. Males were almost three times more likely than females to meet the criteria for alcohol abuse and/or dependence. However, that the male-to-female ratio is lowest in the youngest age group among nonblacks suggests that nonblack females may be catching up. This phenomenon does not generalize to black females because the male-to-female ratios in blacks were shown to decrease as a function of age. Possible reasons for the greater discrepancy between male and female rates of alcohol abuse and dependence among younger blacks compared with younger nonblacks include differential age-related role responsibilities or differences in perceived social acceptability of drinking per se between the ethnic groups in the general population.

The overall prevalence estimates and corresponding population estimates of alcohol abuse and dependence presented here do not differ greatly from those for the years 1984 (Williams et al. 1989) or 1988 (Grant et al. 1991), even though these earlier figures were based on diagnostic criteria from the DSM–III (APA 1980) and the DSM–III–R, respectively. The prevalence of DSM–III alcohol abuse and dependence reported by the 1984 National Survey on Alcohol Use was 8.58 percent for the total sample, with an associated population estimate of 15,100,000. The corresponding DSM–III–R prevalence rate for the 1988 National Health Interview Survey was 8.63 percent, representing 15,295,000 Americans. Although these figures are nearly identical to the prevalence of DSM–IV alcohol-related disorders found in the 1992 NLAES sample, caution must be exercised in assuming the stability of these rates between 1984 and 1992. Because definitions of disorders differed among the three surveys, no conclusions can be made concerning the rates of alcohol abuse and dependence over time.

Although the purpose of this Epidemiologic Bulletin is to present the national rates of alcohol abuse and dependence according to the most recent psychiatric classification of alcohol-related disorders (i.e., the DSM–IV), provisions also were made within the NLAES to measure alcohol abuse and dependence by historic diagnostic classifications (i.e., the DSM–III and DSM–III–R). Representation of multiple definitions of alcohol-related disorders will facilitate direct comparisons between the NLAES DSM–III estimates and the DSM–III estimates of the 1984 National Survey on Alcohol Use and between the NLAES DSM–III–R estimates and the DSM–III–R estimates derived from the 1988 National Health Interview Survey. It remains to be seen if trends exist over time in alcohol abuse and dependence. Such trends will become evident once the diagnostic definitions across these surveys are equalized. To this end, a series of reports focusing on trends in alcohol-related disorders between the years 1984 and 1992 currently are being prepared by NIAAA. These reports will present, for the first time, changes in the rates for alcohol abuse and dependence over the last decade.

## Figures and Tables

**Table 1:** Prevalence and Population Estimates^1^ of DSM–IV Alcohol Abuse and Dependence by Age, Sex, and Ethnicity: United States, 1992

	Alcohol Abuse Only			Alcohol Dependence Only			Alcohol Dependence With Abuse			Total Alcohol Abuse and Dependence		
				
Ethnicity/Sex/Age	Prevalence (%)	S.E.	Population Estimate	Prevalence (%)	S.E.	Population Estimate	Prevalence (%)	S.E.	Population Estimate	Prevalence (%)	S.E.	Population Estimate
Nonblack												
Males	4.93	(0.21)	3,928	2.10	(0.14)	1,673	4.30	(0.21)	3,423	11.33	(0.34)	9,024
18–29	10.02	(0.58)	2,031	3.91	(0.34)	792	9.55	(0.55)	1,935	23.48	(0.84)	4,758
30–44	4.81	(0.30)	1,308	2.01	(0.23)	546	4.07	(0.32)	1,107	10.89	(0.47)	2,961
45–64	2.51	(0.31)	521	1.44	(0.22)	300	1.66	(0.25)	346	5.61	(0.44)	1,167
65+	0.60	(0.19)	69	0.31	(0.09)	36	0.30	(0.09)	35	1.21	(0.23)	140

Black Males	2.50	(0.43)	237	2.48	(0.39)	235	3.27	(0.42)	310	8.25	(0.72)	782
18–29	3.97	(0.92)	116	3.92	(0.99)	115	4.44	(1.01)	130	12.33	(1.70)	361
30–44	2.78	(0.78)	96	2.58	(0.65)	89	3.39	(0.71)	117	8.75	(1.21)	302
45–64	1.17	(0.58)	25	1.12	(0.35)	24	2.90	(0.72)	62	5.19	(0.97)	111
65+	0.00	(0.00)	0	0.74	(0.50)	7	0.08	(0.08)	1	0.82	(0.51)	8

Total Males	4.67	(0.19)	4,165	2.14	(0.13)	1,908	4.19	(0.19)	3,733	11.00	(0.32)	9,806
18–29	9.26	(0.52)	2,147	3.91	(0.34)	907	8.90	(0.50)	2,065	22.07	(0.77)	5,119
30–44	4.58	(0.28)	1,404	2.07	(0.21)	635	4.00	(0.29)	1,225	10.65	(0.45)	3,264
45–64	2.38	(0.28)	546	1.41	(0.20)	324	1.78	(0.24)	408	5.57	(0.41)	1,278
65+	0.55	(0.18)	69	0.34	(0.09)	43	0.29	(0.08)	36	1.18	(0.22)	148

Nonblack												
Females	1.62	(0.11)	1,379	1.20	(0.10)	1,019	1.43	(0.10)	1,216	4.25	(0.20)	3,614
18–29	4.29	(0.34)	851	2.60	(0.28)	515	4.10	(0.35)	814	10.99	(0.64)	2,180
30–44	1.58	(0.17)	428	1.23	(0.15)	335	1.13	(0.15)	307	3.94	(0.27)	1,070
45–64	0.42	(0.10)	93	0.68	(0.14)	149	0.35	(0.07)	75	1.45	(0.19)	317
65+	0.04	(0.03)	7	0.13	(0.06)	20	0.12	(0.07)	20	0.29	(0.09)	47

Black												
Females	0.71	(0.16)	84	1.30	(0.21)	153	0.87	(0.18)	102	2.88	(0.32)	339
18–29	1.24	(0.42)	43	1.70	(0.41)	59	0.38	(0.15)	13	3.32	(0.60)	115
30–44	0.98	(0.30)	40	1.18	(0.33)	48	2.02	(0.49)	83	4.18	(0.65)	171
45–64	0.02	(0.02)	0	1.70	(0.52)	45	0.20	(0.12)	5	1.92	(0.54)	50
65+	0.00	(0.00)	0	0.00	(0.00)	0	0.00	(0.00)	0	0.00	(0.00)	0

Total												
Females	1.51	(0.10)	1,463	1.21	(0.09)	1,172	1.36	(0.09)	1,318	4.08	(0.18)	3,953
18–29	3.83	(0.30)	894	2.46	(0.25)	574	3.55	(0.30)	827	9.84	(0.56)	2,295
30–44	1.50	(0.15)	469	1.23	(0.14)	383	1.25	(0.15)	391	3.98	(0.25)	1,243
45–64	0.38	(0.09)	93	0.79	(0.14)	194	0.33	(0.07)	81	1.50	(0.18)	368
65+	0.04	(0.03)	7	0.12	(0.05)	20	0.11	(0.06)	20	0.27	(0.09)	47

Total	3.03	(0.11)	5,628	1.66	(0.08)	3,080	2.72	(0.11)	5,052	7.41	(0.20)	13,760
18–29	6.54	(0.33)	3,041	3.18	(0.21)	1,481	6.22	(0.30)	2,893	15.94	(0.53)	7,415
30–44	3.02	(0.16)	1,873	1.64	(0.13)	1,018	2.61	(0.17)	1,615	7.27	(0.26)	4,506
45–64	1.35	(0.15)	639	1.09	(0.12)	518	1.03	(0.12)	488	3.47	(0.22)	1,645
65+	0.25	(0.08)	75	0.21	(0.05)	63	0.18	(0.05)	55	0.64	(0.10)	193

1 All population estimates are in thousands.

NOTE: Components may not always sum to the totals displayed in the table because of rounding.

**Table 2:** Ratios of Prevalence of DSM–IV Alcohol Abuse and Dependence by Age and Ethnicity: United States, 1992

Ethnicity/Age (years)	Male-to-Female Ratio
Nonblack	
18–29	2.1
30–44	2.8
45–64	3.9
65+	4.2

Black	
18–29	3.7
30–44	2.1
45–64	2.7
65+	—

Total	
18–29	2.2
30–44	2.7
45–64	3.7
65+	4.4

NOTE: Male-to-female ratio is undefined for blacks because of the female rate of 0.0.

## Footnotes

1 For a definition of this term and others used in this article, see glossary on p. 244.

## GLOSSARY

Cluster sampling: A sampling method in which each sampling unit is a collection of persons, units, or elements of interest.
Oversampling: A sampling technique used to bolster the numbers within low-prevalence subgroups of the population in order to achieve adequate numbers suitable for statistical analysis.
Primary sampling units: Comprehensive, mutually exclusive categories, consisting of all persons, units, or elements of interest, usually identified in the first stage of a multistage sampling design. For example, *primary sampling units* can consist of geographic regions of the United States (e.g., cities) defined in terms of sociodemographic criteria.
Selected with probability: This typically refers to the selection of sampling units according to predetermined probabilities. For example, *primary sampling units* may be selected that have probabilities proportional to size.
Selected with certainty: This typically refers to the selection of sampling units with a probability of 1.0. For example, if *primary sampling units* are designated to be selected in proportion to their size, it follows that the largest of the units will be selected with certainty.
Simple random sample: A method of drawing samples such that each person, element, or unit has an equal probability of being selected.
Stratification: The classification of all persons, units, or elements of interest into comprehensive, mutually exclusive categories.
Variance estimation procedures: A technique that allows estimation of the amount of dispersion around a measure of data, such as a percentage or mean.
Weighted percentage: Percentages that have been adjusted to account for all aspects of the sample design (e.g., differential rates of selection, *over-sampling)*.
